# The Impact of Audio-Visual, Visual and Auditory Cues on Multiple Object Tracking Performance in Children with Autism

**DOI:** 10.1177/00315125231187984

**Published:** 2023-07-15

**Authors:** Lily Hughes, Niko Kargas, Maximilian Wilhelm, Hauke S. Meyerhoff, Julia Föcker

**Affiliations:** 1 School of Psychology, College of Social Science, 120994 University of Lincoln, Lincoln, UK; 2 Center for Psychotherapy Research, 155992 University Hospital Heidelberg, Heidelberg, Germany; 3 38877 University of Erfurt, Erfurt, Germany

**Keywords:** attention, multisensory perception, multiple object tracking, neurodiversity, autism

## Abstract

Previous studies have documented differences in processing multisensory information by children with autism compared to typically developing children. Furthermore, children with autism have been found to track fewer multiple objects on a screen than those without autism, suggesting reduced attentional control. In the present study, we investigated whether children with autism (n = 33) and children without autism (n = 33) were able to track four target objects moving amongst four indistinguishable distractor objects while sensory cues were presented. During tracking, we presented various types of cues - auditory, visual, or audio-visual or no cues while target objects bounced off the inner boundary of a centralized circle. We found that children with autism tracked fewer targets than children without autism. Furthermore, children without autism showed improved tracking performance in the presence of visual cues, whereas children with autism did not benefit from sensory cues. Whereas multiple object tracking performance improved with increasing age in children without autism, especially when using audio-visual cues, children with autism did not show age-related improvement in tracking. These results are in line with the hypothesis that attention and the ability to integrate sensory cues during tracking are reduced in children with autism. Our findings could contribute valuable insights for designing interventions that incorporate multisensory information.

## Introduction

Autism Spectrum Disorder (ASD; hereafter termed ‘autism’) is a neurodevelopmental disorder defined by the presence of atypical social and communication capacities alongside the presence of repetitive behaviors and specialized interests (American Psychiatric Association [APA], 2013). The Diagnostic and Statistical Manual of the Mental Disorders, Fifth Edition (DSM-5) includes atypical sensory processing as a key characteristic of ASD (APA, 2013), with sensory dysfunction reported in 69–95% of individuals with autism (e.g., Baker et al., 2008; Tomchek & Dunn, 2007). Despite only recently being recognized as a defining feature, sensory differences across multiple modalities have been reported widely with autism over recent decades (see Feldman et al., 2018 for review). Moreover, though not part of current diagnostic criteria, abnormal attentional capacities are prevalent in autism (Keehn et al., 2010). Given that an individual’s interaction with a dynamic multisensory world is facilitated entirely through perceptual processing and that perceptual development is mediated by an individual’s ability to integrate multisensory information into coherent wholes, an alteration in the integration of sensory input (in several modalities) may explain the atypical behavioral expressions characteristic of individuals with autism (Bahrick & Todd, 2012; Iarocci & McDonald, 2006).

### Attentional Mechanisms in Children with Autism

Research on attentional functioning in autism has revealed difficulties in shifting attention (Landry & Bryson, 2004), widening of attention from local to global information (Mann & Walker, 2003) and processing multiple features from visual scenes (O’Hearn et al., 2011). Several studies have investigated the ability in children with autism to track multiple moving objects (Koldewyn et al., 2013; O’Hearn et al., 2011). Multiple object tracking (MOT; see Meyerhoff & Papenmeier, 2020, for a freely available demonstration) is an experimental, perceptual-cognitive paradigm that combines features of visual motion perception, low-level attentional processing, and high-level cognitive components; and the MOT task has been identified as a prototypical task for assessing the efficiency of attentional processing (Meyerhoff et al., 2017).

The MOT task requires individuals to track on a screen a specific number of identical target objects (moving on different trajectories) amongst several distractor objects (see Meyerhoff et al., 2017 for review). Participants are asked to identify the target objects, and their tracking accuracy typically serves as an outcome variable. Some studies have observed that 5–12-year-old children with autism show reduced tracking accuracy compared to children without autism, irrespective of motion speed (Koldewyn et al., 2013; O’Hearn et al., 2013). Other studies have shown that strategies such as grouping target objects improves tracking abilities, not only in children without autism (O’Hearn et al., 2013) and neurotypical adults (Erlikhman et al., 2013; Scholl et al., 2001) but also in children with autism (O’Hearn et al., 2013). For instance, when children were asked to track target objects that were coupled with another target in both the so-called “grouping helps” condition or with a distractor in –the so called “grouping hurts” condition (p.12 and following), children with autism were able to track more objects in the target grouping condition compared to the distractor grouping condition (see also Evers et al., 2014).

#### Multisensory Processing in Children with Autism

When navigating through a complex visual environment, individuals are required to simultaneously track, examine, and integrate multiple moving objects. For example, walking through a local park requires distributing attention and spatially integrating moving entities (e.g., pedestrians, bikes, and animals). It is therefore unlikely that social scenarios would be exclusively centered around the visual modality. As in previous research, to investigate the attentional capacity of individuals with autism using MOT-tasks, we focused on how both unisensory cues (visual or auditory) and multisensory cues (audio and visual cues) impact tracking performance (attentional capacities) in children with autism.

Research on adults without autism has shown that motion dynamics, such as changes of direction (Fencsik et al., 2007) and speed (Meyerhoff et al., 2017), impair multiple object tracking performance. The introduction of a visual “identity” to the moving objects has revealed that visual information has a positive impact on tracking abilities. For example, a brief color change to the distractor objects during instants of spatial proximity with target objects improved tracking accuracy (Bae & Flombaum, 2012). This implies that adults without autism can use the identifying color information to track objects across instants when confusion errors can transpire (Drew, et al., 2013); additionally, color contributes to object correspondence (Papenmeier et al., 2014). More recently, Föcker et al. (2022) investigated whether auditory and audio-visual cues improve multiple-object tracking for adults. In this task, participants were asked to track five target objects amongst five distractor objects. During tracking, visual, auditory, and audio-visual cues were delivered when objects bounced against the inner central circle; and they elicited sensory cues that updated the target-relevant object information and, thus, improved tracking performance. Both auditory and visual cues improved tracking accuracy, compared to the absence of any cues.

Multisensory integration (MSI) is the ability to combine information across different sensory modalities, and it has been found to improve performance in low-level (target detection) and high-level (speech perception) tasks more than with only unisensory cues (Ainsworth et al., 2021; Molholm et al., 2004). Importantly, MSI is one facet of sensory function that appears to be disrupted in autism (Feldman et al., 2018). Some studies have investigated MSI in complex socially related speech tasks. Overall, these findings demonstrate that children with autism do not benefit from the addition of visual cues during speech tasks (audio-visual integration), indicating that the interaction of multiple senses is disrupted in children with autism (Foxe et al., 2015; Irwin et al., 2011; Smith & Bennetto, 2007). These findings have been replicated in studies using the McGurk illusion, in which an auditory stimulus is mismatched with a visual stimulus resulting in a third phoneme being perceived that was not inferred from the auditory or visual stimuli (McGurk & MacDonald, 1976). However, children with autism have been found to be less susceptible to this illusion, indicating that, relative to children without autism, they have weaker audio-visual integration during phonemic-level inputs (Bebko et al., 2014; DePape et al., 2012; Irwin et al., 2011; Mongillo et al., 2008; Stevenson et al., 2014). These findings are not consistent across the literature, however (see Iarocci et al., 2010; Saalasti et al., 2011; Woynaroski et al., 2013). For instance, researchers observed that, when lip reading abilities were controlled, children with autism no longer showed impairments in multisensory integration; this suggests that children with autism have a reduced reliance on visual information, even though they show intact multisensory integration abilities (Williams et al., 2004).

It might be argued that individuals with autism might focus their attention on the main task to track the moving objects, failing to integrate other sensory signals during tracking (e.g. the auditory and audio-visual cue). This question is relevant, as understanding how sensory cues affect attentional processing in this population may provide further insight into higher-order impairments that are associated with the condition, such as social communication deficits. Using an object tracking task that integrates sensory cue information within a research paradigm will help determine whether differences in sensory and attentional functioning are primary areas of impairment in autism, or whether differences between these individuals and others emerge in more complex socially related scenarios.

For instance, the Sound Induced Flash Illusion (SiFi; Shams et al., 2002) occurs when two visual flashes are presented at the same time as an auditory beep (resulting in the visual illusion of a singular flash). Children with autism are reported to be less susceptible to this illusion compared with children without autism, indicating that these children have less efficient MSI (Stevenson et al., 2014). However, other studies revealed that adolescents and adults with and without autism are equally susceptible; indicating that MSI may depend on the developmental trajectory of the individuals assessed (Van der Smagt et al., 2007; Bao et al., 2017). Moreover, the Pip-and-Pop effect (Van der Burg et al., 2008) indicates that an auditory cue that is simultaneously presented with a color change on a target can normally improve visual search. The temporal synchrony has seemed to enable supramodal binding by stimulating the integration of auditory stimuli through inducing the concurrent perception of a visual stimulus. Thus, this supramodal binding attracts attention in a bottom-up fashion. However, in individuals with autism, reaction times towards the visual stimuli do not improve when an auditory component is added (Collignon et al., 2013), suggesting that children with autism only focus on the main task and ignore the auditory signal. Accordingly, throughout our present experiment, we hypothesized that, for children without autism, auditory cues may be effective in the (re)identification of visual targets by stimulating visual attention towards the target object, while, for children with autism, auditory cues may not have the same performance benefit. Considering that previous investigators have shown that performance during some tasks is not improved through the addition of sensory cues from another modality for children with autism, we aimed to extend those findings to a visual MOT task in which we could present simultaneous sensory cues (auditory, visual, audio-visual cues) during tracking to allow the reidentification of tracked target objects. We also sought to assess whether audio-visual interactions would alter object correspondence in children with autism, considering that information from numerous sensory modalities need to be integrated and has been found to improve performance in low-level and high-level tasks relative to unisensory cues (Ainsworth et al., 2021) in individuals without autism. Therefore, we examined whether auditory, visual, and audio-visual cues impact tracking performances equally or differentially in children with and without autism. Like Föcker et al. (2022), we presented visual, auditory and audio-visual cues during tracking, and we asked participants to keep track of four target objects among four distractor objects. Previous investigators demonstrated that perceptual grouping strategies improved MOT performance (Evers et al., 2014). To understand whether children’s perceptual grouping abilities might predict their MOT performance, we also asked all children to perform the Navon task (Navon, 1977). The Navon task consists of a global letter shape comprised of local letters, and others have shown that children with autism show local, rather than global processing (Muth et al., 2014, for a review).

### Present Study

**Figure 1.**
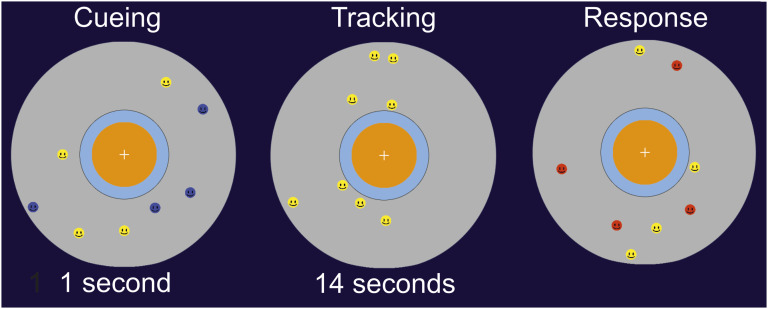
Illustration of the Experimental Task. *Note:* In the Cueing phase, the four targets can be distinguished from four distractors by the blue color. During the tracking phase, the target objects are visually indistinguishable from the distractors. During this phase, only the target objects bounce against the inner orange color and elicit a sensory cue (visual, auditory, audio-visual) or no cue. In the response phase following the object movement, the participants are asked to indicate the target objects (and guess when uncertain).

To investigate the hypothesis that multisensory cues might not facilitate the (re)identification of target objects for children with autism in the same manner as for children without autism, we used a MOT task in which auditory, audio-visual and visual cues were presented when target objects bounced off an inner circle in the tracking area (see Figure 1).

Considering that individuals with autism have been found to allocate attention to more local features of visual tasks, we used a version of the Navon task (Navon, 1977) in which global letters are made up of local smaller letters to measure whether children with autism subconsciously allocate their attention towards local features; this could influence their ability to track multiple objects as they might be less apt to apply a grouping strategy than children without autism and might be less apt to use sensory cues to improve their performances. Additionally, considering that autism is a spectrum condition, we used the parent/caregiver measure, Autism Quotient 10 (AQ-10; Allison, Auyeung & Baron-Cohen, 2012) to assess the extent of any autistic traits in our group of children without autism. We sought to rule out the possibility that the presence of high level autism traits might influence performances of children without a formal diagnosis of autism. We hypothesised that children with autism would process the Navon task by focusing on local features, and in the context of previous studies (e.g., Koldewyn et al., 2013), we also expected that children with autism would show reduced tracking performance, compared to those without autism. Furthermore, we expected that children without autism would profit from some sensory cue information, such as the visual cue (see also adult population who profit most from visual cues). Since difficulties integrating multisensory information have also been reported in children with autism, we expected that they would not profit from the auditory and audio-visual cues to the same extent as children without autism.

## Method

### Participants

Thirty-three children with autism (7 females and 26 males; *M*age = 8.7 years, *SD* = 1.65) and 33 age-matched children without autism (9 females and 24 males; *M*age = 8.7 years, *SD* = 1.65) took part in this experiment. Data on these children’s socio-economic status and ethnicity were not recorded. All children with autism had received a formal diagnosis from a trained clinician in accordance with DSM-5 criteria (APA, 2013). Consistent with the skewed gender ratio for autism in the general population (e.g., Elsabbagh et al., 2012), our sample of children with autism was comprised of more males than females (see Table 1). All participants had normal or corrected-to-normal vision, and the groups were matched in age and education level (school year).

**Table 1.** Descriptive Statistics, Including the Number of Participants in Each Group, and Participants’ Means and Standard Deviations of Age and AQ_10 Scores.

	Autism Present					Autism Absent				
	*N*	*Age*		*AQ-10*		*N*	*Age*		*AQ-10*	
		*M*	*SD*	*M*	*SD*		*M*	*SD*	*M*	*SD*
Male	26	8.9	1.56	7.96	1.75	24	9.0	1.60	2.65	1.89
Female	7	8.1	1.95	7.14	1.46	9	8.1	1.69	1.44	1.01

The effect size of the main effect of visual cue and auditory cue in the previous experiment was *ηp2* = .55 for the Visual Cue condition, and *ηp2* = .26 for the Auditory Cue condition (Föcker et al., 2022). Using the medium effect of f = 0.25 as an estimate for a power analysis (1-ß = .80) based on a repeated measures ANOVA (within-between interaction with two groups and two measurements) with G*Power (Faul et al., 2007, 2009) the power analysis suggests a minimum sample size of 34 participants in total (17 per group). Please note, however, that the effect sizes emerge from a study with healthy adult participants. We therefore recruited 33 children in each group to also consider a possible weaker manifestation of the effect as well as potential dropouts. Please note further that the current study relied on Linear Mixed Effects (LME) analyses to compensate for violations of the ANOVA assumptions. We will return to this discussion in the section of this paper labelled “Limitations and Directions for Further Research.”

Both children with and without autism were mostly recruited in mainstream settings. Additionally, we used opportunity sampling through a post on the social media platform, Facebook, to recruit participants; parents and caregivers could follow an online link to complete the task at home with their child. In the analysis described in the manuscript, we did not exclude any child based on their AQ score, considering that recent findings indicate a moderate correlation (r = .554) between AQ-10 and AQ-50, as well as a poor test-retest reliability (r = .277) of AQ-10 between Time 1 and Time 2 (Cheung et al., 2023). However, in the supplementary material, we present additional analysis in which we excluded two children without autism from the data analysis. These children were excluded because one of them scored higher than 5 on the AQ (score = 7) and the AQ score was not recorded for the other child. However, we retained children with autism who scored less than 6 on the AQ in the analysis, as we consider the clinical formal diagnosis to be a more reliable criterion for classifying autism compared to the AQ score. The pattern of results obtained from this analysis aligns closely with the result pattern reported in the manuscript. Each child who took part gave assent before the experiment and also had written informed consent provided by a parent or caregiver,and the study was approved by the University of Lincoln’s Ethics Committee (ref: PSY2011203).

**Table 2.** Descriptive Statistics, Including Mean and Standard Deviations of Proportion of Correctly Identified Target Objects (MOT scores) in Each Condition and Separately for Children Diagnosed with ASD or Given No Diagnosis.

ASD Diagnosis	Yes		No	
	*M*	*SD*	*M*	*SD*
No cue	0.56	0.17	0.61	0.15
Visual cue	0.56	0.21	0.67	0.15
Auditory cue	0.49	0.14	0.57	0.09
Audio-visual cue	0.56	0.15	0.68	0.16

### Stimuli and Procedure

#### Autism Measure

The Autism Spectrum Quotient (AQ-10) is a brief 10 item parent/caregiver -report screen for autism (Allison, et al., 2012). We asked parents to fill out the AQ on behalf of their child. As our study was conducted on children, a supervising adult who knew the child’s behavioral preferences and tendencies answered the extent to which they agreed with the statements by selecting one of the four response options “Strongly Agree,” “Slightly Agree,” “Slightly Disagree,” and “Definitely Disagree.” The AQ-10 is scored on a dichotomous response format by allocating both “Strongly Agree” or “Slightly Agree” responses a numerical value of 1 and the “Strongly Disagree” and “Slightly Disagree” responses a numerical value of 0. The scores are then summed to provide an overall score out of 10. Previous research indicates that a score of 6 or higher is an indicator that an individual may be autistic (Allison et al., 2012).

#### Navon Task

**Figure 2.**
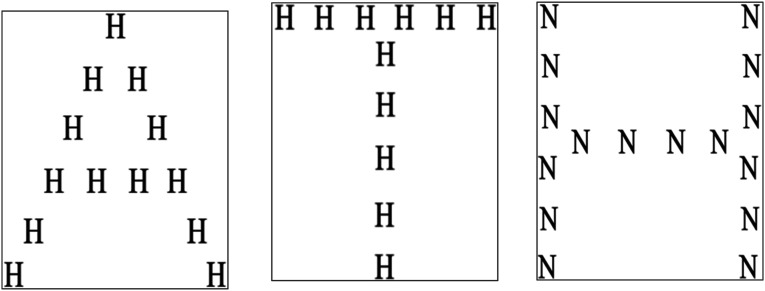
Illustration of the Navon Task (i.e. “Global” Letters are Formed out of “Local” Letters).

The Navon task was created with modifications from Booth’s (2006) Navon Similarity Judgement Task. The stimuli consisted of global letters derived from contrasting local letters (see Figure 2). The letters A, F, H, N and T were used at both global and local levels to create 12 stimulus types (Ah, An, Fh, Ft, Ha, Hf, Hn, Ht, Na, Nh, Tf, Th). These letters were selected as they were adequately matched on visual complexity and frequency (Solso & King, 1976; Booth, 2006). The global form measured 5.5 cm in height and 4.5 cm in width. Each local letter was approximately 0.5 cm in height and 0.4 cm in width (see Figure 2).

#### Multiple Object Tracking Task

The experimental MOT task has been adapted on the basis of Föcker et al. (2022) and Green and Bavelier (2006); and it was designed in Unity (Version 2019.11.fl). In this study, the task was presented on a desktop computer or laptop.

The tracking area consisted of a grey circle (diameter: 20 cm, 18.9^o^) with an orange circle (diameter: 5.8 cm, 4.77^o^) positioned in the center of the screen. Blue and yellow smiley faces (diameter: 1 cm, 0.95^o^) moved within the grey circle, bouncing off the boarder of the orange circle. When the *target objects* (originally blue smiley faces) bumped against the inner part of the orange circle, a sensory cue wase elicited. A color change from yellow to blue (V, duration = 0.15 seconds) represented the visual cue, and a tone (A, 440 Hz, duration = 0.15 seconds) represented the auditory cue. Both a color change and a tone represented the audio-visual cue (see Figure 1). In the control cue condition, neither a color change nor sound was elicited when the target object bounced off the inner orange circle. The movement of the smiley faces followed Newtonian mechanics on a 2D plane. The preliminary direction of object movement was random (moving at 2 pixels per frame; 3.6 degrees per second). Each target struck the inner orange circle once per trial, moving towards the circle in a straight line (speed: 2 pixels per frame). This bouncing effect occurred every 2.55 seconds.

Children were seated directly in front of a laptop or desktop computer on which the stimuli were presented. On the screen, they were instructed as follows by the gatekeeper supervising the experiment: “*Looking at this image, which letter do you see here first? There is no right or wrong answer, as either could be right. Try not to take too long to think about it, just quickly choose the one you see first”.*

Once the child was ready, the gatekeeper clicked ‘next’ which triggered the first of the 12 stimuli. Children were asked to respond verbally as quickly as possible as to which letter they perceived first. The gatekeeper then used the keyboard on the desktop computer or laptop to type in the answer provided before moving onto the next image.

At the start of the first trail, four blue target smiley faces and four yellow distractor smiley faces start by moving on the screen for 1000 ms (Cueing phase). Children were asked to keep track of the blue target smiley faces while ignoring the yellow distractors. The blue targets turned yellow after 1000 ms (tracking phase) making them indistinguishable from others. When target smiley faces bounced off the inner orange circle, an auditory cue, visual cue, audio-visual cue, or no-cue was elicited (see Figure 1).

Once the targets had stopped moving, children were asked to point out to the gatekeeper the four smiley faces they thought were the original blue faces. Children were prompted to guess if they did not know. The selected smiley faces turned red when clicked; participants could correct their initial judgement by unmarking the selected face. The study consisted of eight blocks: auditory cue (A), visual cue (V), audio-visual cue (AV) an no cue (NC). Each block included one of these conditions and was repeated twice meaning children were trained on each experimental block. The order of conditions was counterbalanced across participants: (1) AV, V, NC, A; (2) NC, A, V, AV; (3) V, AV, A, NC; (4) A, NC, AV, V.

### Data Analysis

The proportion of the participants’ correct tracking accuracy responses was predicted using linear mixed effects models including *Visual Cue* (Present, Absent), *Auditory Cue* (Present, Absent), *Age* (6–11 years) and *Group* (children with and without autism) as fixed effects and subjects as a random effect. The advantage of using LME models compared to analyses of variance (ANOVAs)is in handling missing data (e.g., unbalanced data sets) and dealing with small sample sizes. Furthermore, LME models have advantages in their ability to model non-linear, individual characteristics (Krueger & Tian 2004). Additionally, they allow for multiple observations from the same participant and deal with non-normally distributed and skewed data. Therefore, these models were preferred over traditional ANOVAs. Linear Mixed effects models were calculated using the software package SPSS Statistics version 27. Plots were created in R (Version 4.2.2; R Core Team, 2022). Data files have been shared on OSF (https://osf.io/a2qbd/). For the models, *p* values of overall effects were determined using conditional *F* tests using a Type III ANOVA, with *p* < .05 considered statistically significant.

## Results

### Main Analysis

**Figure 3.**
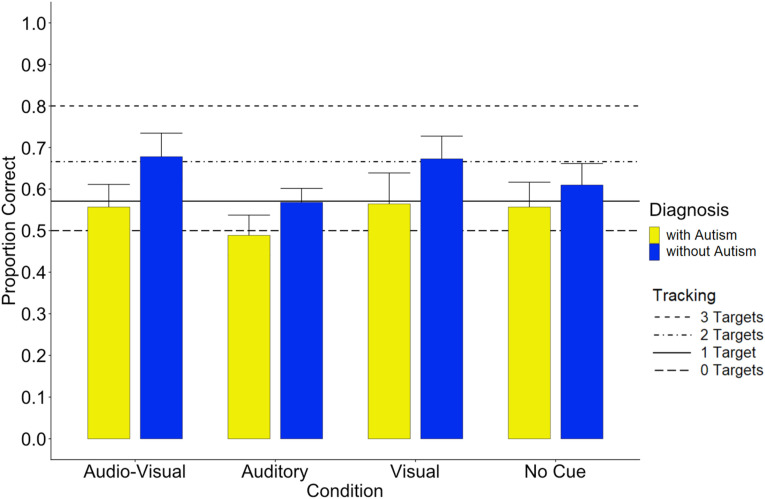
Proportion of Correct MOT Performances in the Group without Autism (Control), and in the Autism Group (AG), Shown Separately for the Different Conditions (Audio-Visual, Auditory, Visual and No Cue). *Note.* The horizontal lines depict the tracking performance levels to be expected, according to Hulleman (2005) at tracking capacities of one, two, and three targets, with zero targets indicating chance level.

There was a significant main effect of *Visual Cue, F*(1,162) = 10.67, *p* = .001, with a higher proportion of correct target detection in the overall sample when visual cues were present (*M* = .62; *SE* = .015, 95% CI [.587; 646]) compared to when visual cues were absent (*M* = .56; *SE* = .015; 95% CI [.527; .587]; see also Figure 3). Moreover, the main effect of *Group* was significant, *F*(1,54) = 13.787, *p* < .001, with a lower proportion of correct target detection among children with autism (*M* = .54; *SE* = .017; 95% CI [.508; .576], Figure 2) compared to children without autism (*M* = .63; *SE* = .017, 95 CI [597; .665]). The main effect of *Age* was not significant, nor were any interactions, including the factor Age significant, (main effect of age: *F*(5,54) = .329, *p* = .893; interaction with age: all *p*s > .178). All other main and interaction effects were not significant (all *ps* > .05). Descriptive statistics are reported in Table 2.

#### Exploratory Analysis

Although there were no interactions in the main analysis, visual inspection of Figure 2 seemed to suggest that children with autism might show poorer performance, especially in the sensory cue conditions. We therefore performed separate LMEs for each group, including the factors *Age*, *Visual Cue (present, absent)*, and *Auditory Cue (present, absent)* as fixed effects, and the factor*, subject,* as random effect.

For children with autism, we found no significant main or interaction effects between the factors *Visual Cue* and *Auditory Cue* (main effect of *Visual Cue*: *F*(1,81) = 1.839, *p* = .179 main effect of Auditory Cue: *F*(1,81) = 1.122, *p* = .293; interaction between *Visual and Auditory* Cue: *F*(1,81) = .606, *p* = .493). Moreover, the main effect of *Age* and the interactions that included the factor, *Age,* were also not significant.

**Figure 4.**
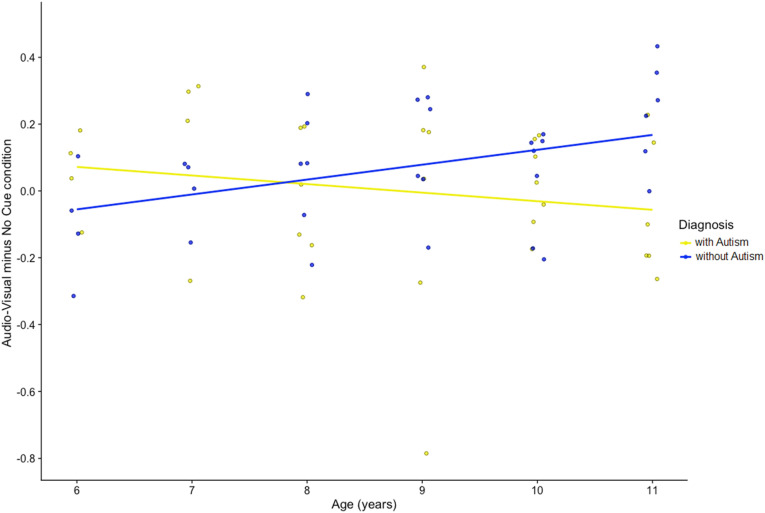
Correlations Between Age and Proportion of Correct Target Identification of the Difference Scores of the Audio-Visual Condition Minus the No Cue Condition in Children with Autism (yellow) and Without Autism (blue).

For children without autism, the main effect of *Visual cue* was significant, *F*(1,81) = 15.28, *p* < .001. Further, visual cues improved tracking performance in this group relative to the absence of any cues (*Visual cues* present: .670, SE = .02; 95% CI [.63; .71]; *Visual cues* absent: .592, SE = .02; 95% CI [.55; .63]). Moreover, the interaction between *Auditory cues*, *Visual cues* and *Age* was significant, *F*(5,81) = 2.504, *p* = .037. This three-way interaction emerged from an increasing use of sensory cues with increasing age in children without autism (r = .399, *p* = .021; N = 33, see Figure 4).

### Global versus Local Perception

To investigate local versus global perception in children with and without autism, we calculated a Chi Square test. There was a significant relationship between the Navon task and a diagnosis of autism, χ^2^ (1) = 15.66, *p* < .001, N = 66. Among children with autism 23 of 33 children indicated local perception of the Navon letter, whereas 26 of 33 children without autism indicated the global letter shape.

## Discussion

Our aim in the current study was to investigate whether tracking performance in children with autism benefits from additional sensory cues during a multiple object tracking task. Our results demonstrated that children with autism tracked fewer target objects successfully compared to those without autism across all sensory cue conditions. Furthermore, children with autism showed a more local processing strategy compared to those without autism.

Our findings are consistent with previous research, suggesting that children with autism track fewer objects in a MOT task than their neurotypical peers (Koldewyn et al., 2013). However, we extended these previous findings by investigating the influence of sensory cues on attentional capacities. Beker et al. (2018) proposed that both these functions are significant in the development of higher-level functions, including social interaction and communication. For instance, having a conversation not only requires tracking multiple non-verbal cues simultaneously (multiple objects), but also requires processing and integrating multiple sensory inputs (audio and visual information).

We aimed to understand whether unisensory cues (visual *or* audio) and multisensory cues (audio *and* visual) that coincide with changes in direction during the tracking of multiple objects impact performance in children with autism. In individuals without autism, visual cues were found to be effective during tracking target objects, compared to no-cues, which is in line with previous studies, including one with an adult population (Bae & Flombaum, 2012; Föcker et al., 2022). However, auditory cues were not effective in the multiple object tracking performance of children without autism compared to adults (Föcker et al., 2022). Please note that we did not find the corresponding interaction involving the diagnosis of autism to be significant; but, nevertheless, our study provides some initial evidence for further exploration of these potentially distinct patterns of results. Indeed, there might be different reasons why children (irrespective of the autism diagnosis) do not include auditory cues when tracking multiple moving objects. Perhaps the task difficulty in our study was too high to unfold the enhancing effect of the auditory cues. A low load task (i.e., two target objects compared to four) might improve the ability to integrate the direction change of the object with the sound of the object. On the other hand, several investigators observed that optimal multisensory integration has a protracted time course of development, maturing only at around 8–10 years (Gori et al., 2008; Nardini et al. 2008; Petrini et al., 2014). For example, Gori et al. (2008) observed a unisensory dominance in younger children, who relied more on haptic cues when they were asked to discriminate the height of two sequentially presented blocks, whereas optimal visual-haptic integration skills were observed in 8–10 years old children. Thus, it might be argued, that the ability to integrate the object’s auditory cues and visual direction changes increases with age. In line with this hypothesis, our exploratory evidence suggests that the ability to use multisensory cues during tracking increases with age in typically developing children but might be guided by the visual modality at a younger age (cross-sensory calibration hypothesis). According to this cross-sensory calibration hypothesis, the more accurate sensory modality serves to “teach” or calibrate the others. As vison is the most dominant sense for localizing objects it might be more informative than other sensory modalities.

Previous behavioral studies have yielded inconsistent results in atypical multisensory processing among participants with autism (see Beker et al., 2018 for review). However, this research has revealed a decrease in the level of automatic integration of sensory information in children with autism relative to their peers without autism (Brandwein et al., 2012); individuals with autism do not benefit from multisensory or unimodal visual cues during speech perception tasks (Smith & Bennetto, 2007; Irwin et al., 2011; Foxe et al., 2015; Bebko et al., 2014). Even though individuals with autism are less successful at tracking overall, compared to typically developing peers, we discovered a main effect of visual cues, showing that these cues are beneficial during reidentification of target objects. Furthermore, Collignon and colleagues (2013) identified significant group differences in adults with autism during auditory-based facilitation; however, they found superior performance in all participants during unisensory visual conditions. This is consistent with enhanced visual search performances found in participants with autism (O’Riordan et al., 2001) and increased ability to detect embedded figures (Shah & Frith, 1983).Weak Central Coherence theory (WCC; Happé & Frith, 2006) and Enhanced Perceptual Functioning theory (EPF; Mottron et al., 2006) both propose that individuals with autism are predisposed toward reliance on local information. We used a version of the Navon task (1977) to measure whether participants naturally focused on the local or global visual elements, and we found a significant relationship among participants between having autism and naturally processing local elements on the Navon task. Thus, individuals with autism focused more on the local visual elements of the task, perhaps explaining why visual cues were used to improve the tracking of target objects; this presumed bias towards visual information may have interfered with our results.

The lack of bimodal facilitation in our participants with autism may relate to their reduced efficacy for integrating local information into global wholes. Similarly, this deficit may explain why their performance was less successful than that of children without autism; the natural tendency to allocate attention towards detailed versus holistic image information in the autism group suggests that the facilitation of sensory cues is less likely to have enhanced performances in this group in the same manner as in the group without autism. However, we did not find a correlation between Navon test performance and MOT performance, suggesting that the underlying processes involved in these tasks may be different.

An alternative explanation for an overall lower performance in participants with autism might be explained by predictive coding theory which suggests that there is a weakness in these individuals in their ability to compare incoming bottom-up sensory information with a top-down prediction of the world (Chan et al., 2016). Individuals with autism appear to overestimate prediction errors such that small disparities in sensory input may be processed as more valuable than their face value (i.e., prediction errors cannot be ignored which attracts further processing and overly sensitive responses to external input). For these participants, more attention may have been directed towards the distractor objects, resulting in fewer target objects being tracked. Importantly, this is known to occur only when multiple cues are perceived (Burack, 1994), suggesting that additional sensory cues from a different sensory modality (i.e., auditory *and* audio-visual) in the MOT task (i.e., visual task) act as additional distractor elements during tracking (beyond the moving visual stimuli in the task), leading to worse performances than those of peers without autism.

### Limitations and Directions for Further Research

Given that we employed Linear Mixed Effects (LME) models in our analysis, it is important to interpret the sample size calculations reported in our manuscript with caution.

Based on the finding that MOT performance relates to enumeration and mathematics abilities in children (Anobile et al., 2013; Steele et al., 2012; Trick et al., 2012; Wilmer et al., 2016), additional cognitive and educational measurements could be collected in future studies, such as mathematics and language performance. These additional measurements could also shed light on the exact cognitive differences in children with autism and children who have not been diagnosed with autism. Future studies could also include additional measurements of multisensory integration, such as the McGurk effect (McGurk & MacDonald, 1976) to relate those to the ability of sensory cue integration in the object tracking task.

From a methodological point of view, the MOT task could include a staircase procedure to identify the exact capacity of how many objects participants are able to track in the different sensory cue conditions. In line with a previous review, it might be argued as well that future interventions could focus on multisensory integration to improve higher order cognitive functions such as language (see Baum et al., 2015).

## Conclusion

On complex attentional tasks (MOT), tracking ability has been found to be reduced in individuals with autism (see also Koldewyn et al., 2013; O’Hearn et al., 2013). In this study, across different conditions in which sensory cues were added, children with autism tracked fewer target objects than did their age-matched controls without autism. Our exploratory analysis suggested that this might be especially pronounced in task conditions with sensory cues. Cross-sensory calibration as well as impaired multisensory integration and predictive coding might all provide possible explanations for these effects in participants with autism. Our results further substantiate the hypothesis that, in addition to reduced ability to track moving objects on a screen, the ability to use different sensory cues during multiple object tracking is reduced in children with autism compared to peers without autism. This information is important as it could guide the development of person-centered multisensory learning strategies for children with autism.

## Supplemental Material

Supplemental Material - The Impact of Audio-Visual, Visual and Auditory Cues on Multiple Object Tracking Performance in Children with Autism Click here for additional data file. Supplemental Material for The Impact of Audio-Visual, Visual and Auditory Cues on Multiple Object Tracking Performance in Children with Autism by Lily Hughes, Niko Kargas, Maximilian Wilhelm, Hauke S. Meyerhoff and Julia Föcker in Perceptual, and Motor Skills
