# Supplementary material for: The Impact of Audio-Visual, Visual and Auditory Cues on Multiple Object Tracking Performance in Children with Autism
Source: Percept Mot Skills. 2023 Jul 15;130(5):2047–68. doi: 10.1177/00315125231187984 (PMC10552336; doi:10.1177/00315125231187984)
Supplement: Supplemental Material - The Impact of Audio-Visual, Visual and Auditory Cues on Multiple Object Tracking Performance in Children with Autism [file sj-pdf-1-pms-10.1177_00315125231187984.pdf]

Supplementary Material:

Main Analysis

In the following analysis, we excluded two children without autism from the data analysis. These children were excluded because one of them scored higher than 5 (AQ score = 7) on the Autism Quotient (AQ), and the AQ score was not recorded for the other child. However, we retained children with autism who scored less than 6 on the AQ in the analysis, as we consider the diagnosis to be a more reliable criterion for classifying autism compared to the AQ score.

There was a significant main effect of *Visual cue*, $F(1,156) = 9.321$, $p = .003$, with a higher proportion of correct target detection in the overall sample when visual cues were present ($M = .62$; $SE = .016$, 95% CI [.585; 649]) compared to when visual cues were absent ($M = .56$; $SE = .016$; 95% CI [.527; .589]). Moreover, the main effect of *Group* was significant, $F(1,52) = 13.006$, $p = .001$, with a lower proportion of correct target detection among children with autism ($M = .54$; $SE = .018$; 95% CI [.509; .576]) compared to children without autism ($M = .63$; $SE = .017$, 95 CI [596; .670]). The main effect of Age was not significant, nor were any interactions, including the factor Age significant, (main effect of age: $F(5,52) = .315$, $p = .902$; interaction with age: all *p*s > .078). All other main and interaction effects were not significant (all *ps* > .05).

Exploratory Analysis

Although there were no interactions in the main analysis, visual inspection of Figure 1 seemed to suggest that children with autism might show poorer performance, especially in the

sensory cue conditions. We therefore performed separate LMEs for each group, including the factors *Age*, *Visual Cue (present, absent)*, and *Auditory Cue (present, absent)* as fixed effects, and the factor, *subject*, as random effect.

For children with autism, we found no significant main or interaction effect between the factors *Visual Cue* and *Auditory Cue* (main effect of *Visual Cue*: $F(1,81) = 1.839$, $p = .179$ main effect of Auditory Cue: $F(1,81) = 1.122$, $p = .293$; interaction between *Visual and Auditory* Cue: $F(1,81) = .606$, $p = .493$). Moreover, the main effect of *Age* and the interactions that included the factor, *Age*, were also not significant.

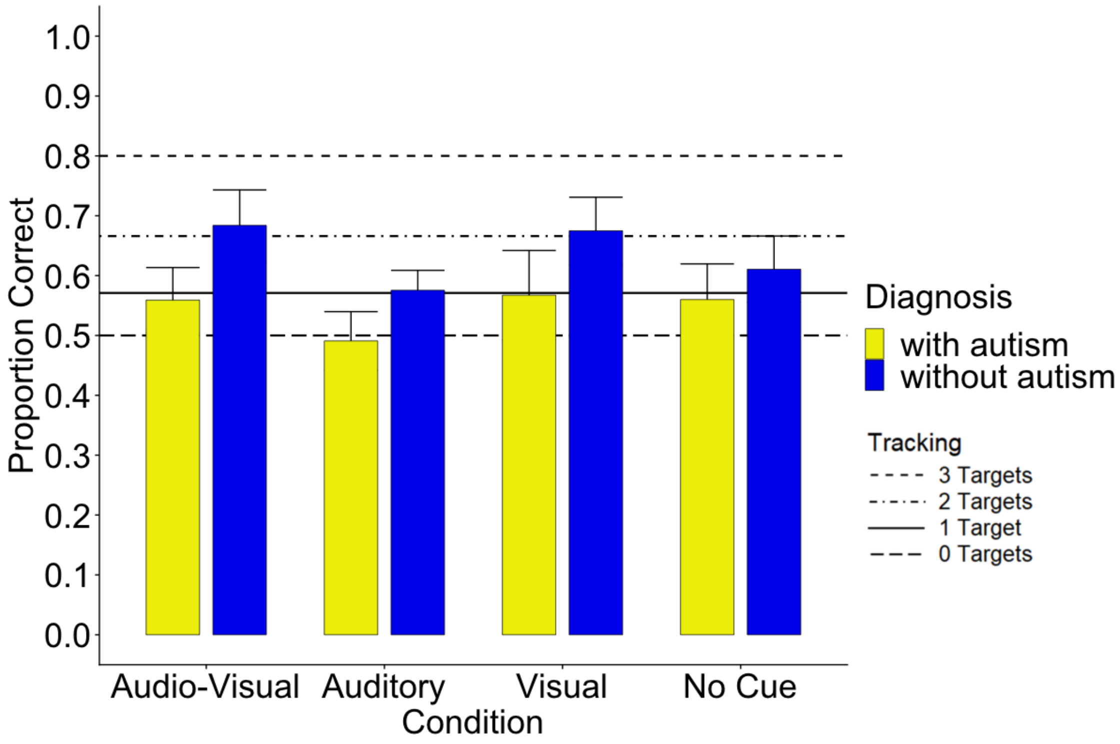

Fig. S1: Proportion of Correct MOT Performances in the Group without Autism (Control), and in the Autism Group (AG), Shown Separately for the Different Conditions (Audio-Visual, Auditory, Visual and No Cue).

For children without autism, the main effect of *Visual cue* was significant, $F(1,75) = 12.35$, $p < .001$. Further, visual cues improved tracking performance in this group relative to the absence of any cues (*Visual cues* present: .670, SE = .02; 95% CI [63;71;] *Visual cues* absent: .592, SE = .02; 95% CI [.55; 63]). Moreover, the interaction between *Auditory cues*, *Visual cues* and *Age* was significant, $F(5,75) = 3.94$, $p = .008$. This three-way interaction emerged from an increasing use of sensory cues with increasing age in children without autism (r = .395, $p = .028$; N = 31, see Figure 2).

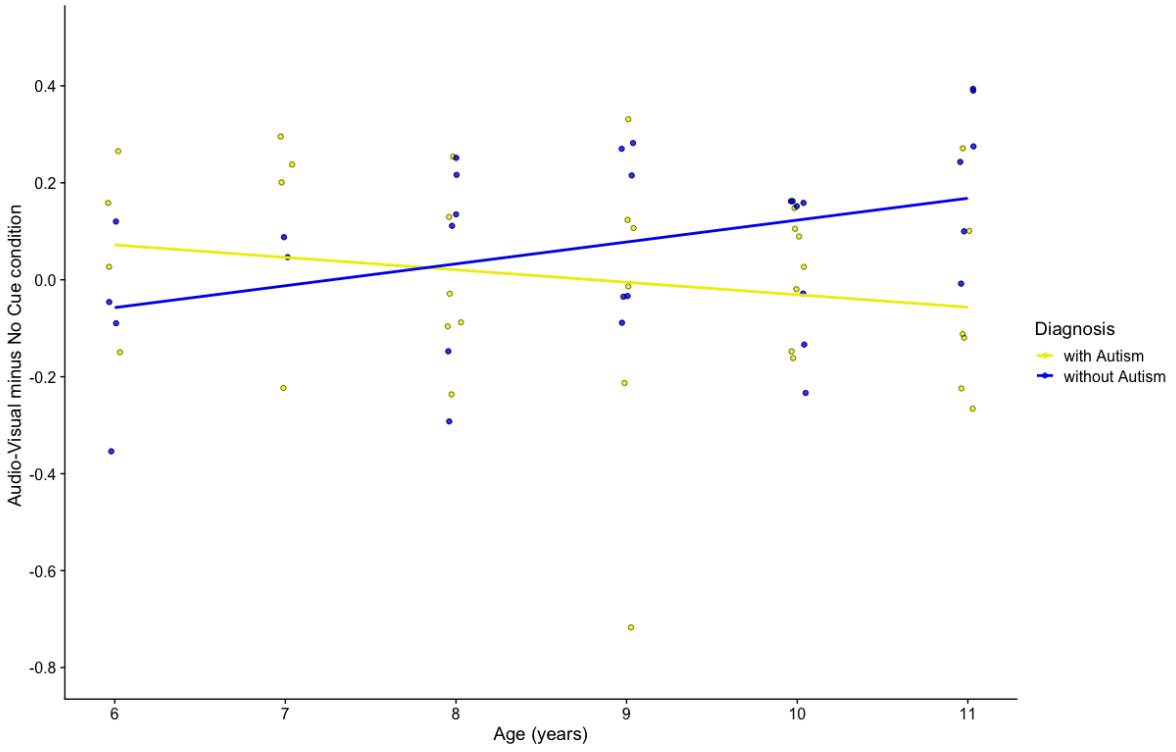

Fig. S2: Correlations Between Age and Proportion of Correct Target Identification of the Difference Scores of the Audio-Visual Condition Minus the No Cue Condition in Children with Autism (yellow) and Without Autism (blue).

Global versus Local Perception

To investigate local versus global perception in children with and without autism, we calculated a Chi Square test. There was a significant relationship between the Navon task and a diagnosis of autism, $\chi2$ (1) = 16.34, p <.001, N = 64. Among children with autism 23 of 33 children indicated local perception of the Navon letter, whereas 25 of 31 children without autism indicated the global letter shape.
